# Infection of Norway spruce by *Chrysomyxa rhododendri*: ultrastructural insights into plant–pathogen interactions reveal differences between resistant and susceptible trees

**DOI:** 10.1093/treephys/tpaf066

**Published:** 2025-06-03

**Authors:** Andrea Ganthaler, Carlos Trujillo-Moya, Stefanie Burger, Juraj Hlavaty, Stefan Kummer, Waltraud Tschulenk, Ingrid Walter

**Affiliations:** Department of Botany, University of Innsbruck, Sternwartestrasse 15, 6020 Innsbruck, Austria; Department of Forest Growth, Silviculture & Genetics, Austrian Research Centre for Forests BFW, Seckendorff-Gudent-Weg 8, 1131 Vienna, Austria; VetCore Facility for Research, University of Veterinary Medicine, Veterinärplatz 1, 1210 Vienna, Austria; Unit of Morphology, Center for Pathobiology, University of Veterinary Medicine, Veterinärplatz 1, 1210 Vienna, Austria; VetCore Facility for Research, University of Veterinary Medicine, Veterinärplatz 1, 1210 Vienna, Austria; Unit of Morphology, Center for Pathobiology, University of Veterinary Medicine, Veterinärplatz 1, 1210 Vienna, Austria; VetCore Facility for Research, University of Veterinary Medicine, Veterinärplatz 1, 1210 Vienna, Austria; Unit of Morphology, Center for Pathobiology, University of Veterinary Medicine, Veterinärplatz 1, 1210 Vienna, Austria

**Keywords:** conifer, forest disease, histology, hypersensitive response, pathogen resistance, *Picea abies*, rust fungus, treeline

## Abstract

Infection of Norway spruce (*Picea abies* L.) by the rust *Chrysomyxa rhododendri* is a major problem in European subalpine forests, causing severe defoliation and reduced growth. However, as with most pathogens from high-elevation environments, little is known about the host–pathogen interaction, the associated plant cellular damage and responses, and their differential expression in susceptible and resistant host trees. Here we report on the development of the biotrophic pathogen in the host tissues, from infection by basidiospores to release of aeciospores, by analysing needles at different time points after infection by histology and transmission electron microscopy. Ultrastructural changes in the host cells, ranging from cell reorganization and degradation to the accumulation of secondary compounds, were localized and characterized in both susceptible and a resistant genotype. *Chrysomyxa rhododendri* formed a dense mycelium in the intercellular spaces of the needle mesophyll of susceptible trees, followed by the formation of subepidermal spermogonia and aecia. Symptomatic needle yellowing corresponded to the spatial expansion of the mycelium and was caused by degradation, but not collapse, of the mesophyll cells with chloroplasts. In needles of the enhanced resistant genotype, only few fungal hyphae appeared, but distinct modifications of the cell walls and an accumulation of electron dense material in the intercellular space appeared. In addition, large tannin droplets were observed around fungal structures, indicating an increased accumulation of polyphenols. The findings are consistent with observations on other heterocyclic rusts and with known physiological and molecular responses of infected trees, including a reduced photosynthetic activity, changes in the needle phenolic profile and a local hypersensitive response. Highly resistant trees may be able to limit fungal growth and associated damage by rapidly enhancing structural and chemical barriers in the needle mesophyll.

## Introduction

Stable and healthy forests are of great significance in mountain areas and high-elevation sites, as they provide a wide range of ecosystem services (Häyhä et al. 2015). Particular emphasis must be placed on their importance in protecting settlements, infrastructure and agricultural land from natural hazards such as avalanches, landslides and rockfalls (Bebi et al. 2001). However, forests are increasingly under pressure due to anthropogenic climate change and related drought stress as well as increasing pathogen pressure (Seidl et al. 2017). Increasing pest infestations are partly related to global warming, as higher temperatures tend to provide favourable conditions for the multiplication and spread of harmful organisms, but also to the reduced defensive power of trees under climatic stress. Therefore, it is highly important to increase the knowledge of high-alpine tree pathogens, which are poorly represented in previous research, and those investigations pose a challenge due to lack of cultivation protocols and the impassable subalpine terrain.

Subalpine spruce forests close to the tree line are often damaged by needle rust infections, caused by the fungus *Chrysomyxa rhododendri* ((DC.) De Bary; [=*Chrysomyxa ledi var. rhododendri* (De Bary) Savile, illegitimate later homonym]; Ganthaler et al. 2014). The genus *Chrysomyxa* Unger is a monophyletic group of rust fungi (Uredinales, Basidiomycota) occurring in the boreal forests of the Northern Hemisphere on Pinaceae, with most species alternating with angiosperm hosts in the Ericaceae (Maier et al. 2003). All are obligate parasites with complex or partially shortened life cycles (for a detailed description of morphology, taxonomy and nomenclature of the genus see Berndt (1999) and Crane (2001)). In the European Alps, *C. rhododendri* is widespread but restricted to high-elevation sites, where Norway spruce (*Picea abies* (L.) H. Karst.) co-occurs with the telial host rhododendrons (mainly *Rhododendron ferrugineum* L.).

**Figure 1:**
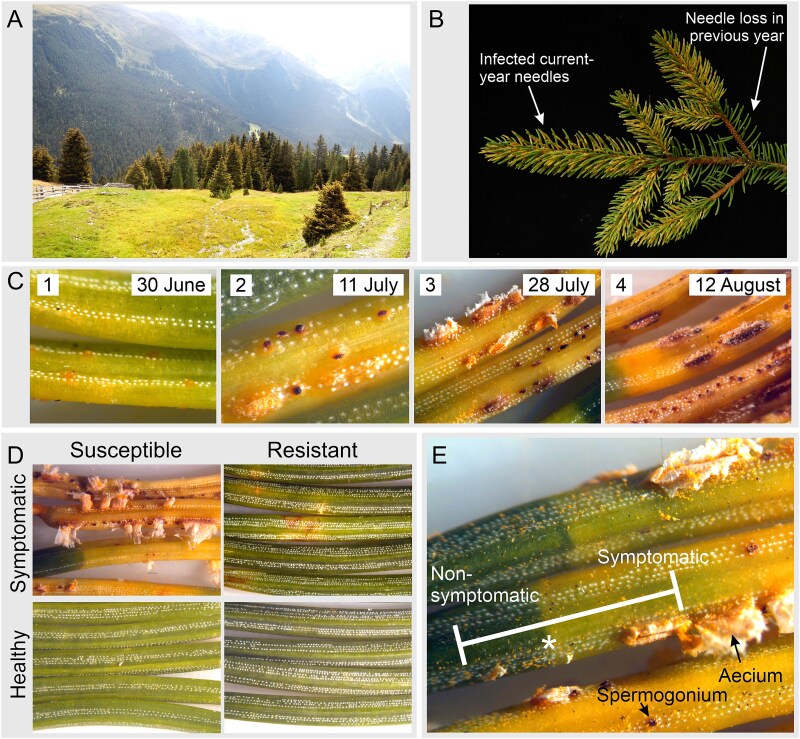
Symptoms of needle rust infection. (A) Subalpine spruce forest in Praxmar (Tyrol, Austria) showing discoloration of the tree crowns due to needle rust infection in August; (B) affected spruce branch with needle rust symptoms in the current-year-foliage and missing needles due to infection in the previous-year foliage; (C) symptom development during the regular sampling (four time points) for one of the susceptible spruce genotypes; (D) symptomatic and non-symptomatic needles of a susceptible and the resistant spruce genotype at the last sampling; (E) infected needles with visible border (asterisk) between non-symptomatic (green) and symptomatic (yellow) parts and spermogonia and aecia.

As is typical for rust fungi, *C. rhododendri* has a rather narrow and specific host range and parasitizes fresh tissues of vigorously growing plants (Cummins and Hiratsuka 1983, Ganthaler et al. 2023). Its life cycle (summarized here according to De Bary 1879, Gäumann 1964, Bennell 1985) begins in spring, when the tough diploid teliospores that overwintered in the evergreen leaves of rhododendrons germinate with simultaneous mitotic division to form a heterobasidium. The haploid basidiospores are released and dispersed by the wind, potentially several kilometres, to Norway spruce trees, where they invade the newly sprouting needles. The infection period is limited to a few weeks between the bud burst (unfolding of spruce needles) and the proceeding needle differentiation, which successfully prevents further infection (Ganthaler and Mayr 2015). After successful infection, a haploid mycelium develops within the conifer needle, causing characteristic yellow bands (Figure 1). The discolouration is probably caused by the carotenoids contained in the fungus and chlorophyll degradation in the plant cells (Pfeifhofer 1989, Bauer et al. 2000). The fungal mycelium then probably forms spermogonia with spermatia and corresponding conception hairs and the contrarian nuclei migrate to aecio complexes. After dikaryotization and mitotic division, aeciospores are formed and released, which can again infect rhododendrons and develop there a dikaryotic mycelium in leaves and stems. The infection can be further amplified between rhododendrons by repeated formation of unicellular urediniospores, before teliospores are formed for overwintering (Crane 2001).

The growth of the fungus in Norway spruce is restricted to individual needles of the current year, but it is still unclear whether the band-shaped yellow discolouration of the needles (Figure 1E) corresponds to the expansion of the mycelium. The tree sheds infected needles in autumn, but can be re-infected in the following spring. Negative effects of needle rust infections on the tree are mainly based on the reduced photosynthetic capacity (in infected needles and due to needle shedding) and strongly depend on the percentage of affected needles. While remaining healthy needles can compensate for small reductions in photosynthetically active tissue by increasing their own photosynthesis (Mayr et al. 2001), repeated high infections in consecutive years lead to almost bare branches. This results in limited biomass accumulation and strongly reduced radial and height growth (Plattner et al. 1999, Mayr et al. 2001). Young plants with a low number of needle age classes have also been observed to die, leading to severe problems in natural regeneration and afforestation in some areas (Plattner et al. 1999, Ganthaler et al. 2014).

The susceptibility of Norway spruce to *C. rhododendri* infection can vary widely within natural populations, and rarely trees with markedly enhanced resistance are found (Mayr et al. 2010). These trees show significantly fewer symptoms than nearby trees, even in years with high airborne spore densities, but the underlying resistance mechanisms are not fully understood (see further details below). One of these trees, already recognized years ago as one of the genotypes with the most pronounced resistance, is PRA-R, which has been the subject of intense research (Mayr et al. 2001, 2010, Ganthaler et al. 2017).

Recent research using RNA sequencing (RNA-Seq) and biochemical analyses suggests that the response of Norway spruce to infection by *C. rhododendri* is locally restricted to the individual needle and is mainly activated between 9 and 21 days after infection (Trujillo-Moya et al. 2020). The response involves a potential isolation of the fungus by a hypersensitive response (HR; Heath 2000) associated with an activation of phenolic pathways. Notably, the infection leads at early stages to an increase in the needle content of flavonoids aglycones, which are potentially fungicidal (Ganthaler et al. 2017). Identified key regulatory genes included those involved in carbohydrate, lipid and secondary compound metabolism (e.g., starch, fatty acids, terpenoids), energy metabolism, translation and signal transduction (Trujillo-Moya et al. 2020). The highly resistant genotype PRA-R was shown to accumulate several flavonoids (mainly kaempferol and taxifolin), stilbenes, abscisic acid and salicylic acid in higher concentrations than highly susceptible genotypes in both healthy and infected needles (Ganthaler et al. 2017). In combination with gene expression analyses, it was concluded that the enhanced resistance may be based on the combination of a strong constitutive defence with a rapid induced response after infection (Trujillo-Moya et al. 2022). However, it has not yet been possible to localize these mechanisms and answer whether they prevent the entry or growth of the rust in the needle or the development of symptoms.

This is partly because our knowledge of the biology of *C. rhododendri*, the infestation process and the plant–fungus interaction are still very limited. Grill et al. (1978) analysed the growth of the related species *Chrysomyxa abietis* in Norway spruce needles in combination with histochemical observations. They showed that the mycelium of this rust fungus remained restricted to the mesophyll and that infection resulted in smaller needle cells with smaller chloroplasts but an enlarged nucleus. Infection also resulted in plasmolysis, increased starch and fat storage and increased activity of the enzymes dehydrogenase, phosphatase and peroxidase in needle cells (Grill et al. 1978). For *C. rhododendri,* there is one ultrastructural study available by Berndt (1999), describing the fungal structures in rhododendron leaves and thus the part of the life cycle in the telial host. However, the fungal development and key processes during infection in the needles of the aecial host, Norway spruce, and the associated plant cellular responses are largely unknown.

The aim of this study was therefore (i) to localize the fungus in the spruce needle and thus to clarify if, when and how hyphae enter the plant cells, which plant tissues are colonized, and if the fungal spread corresponds to the macroscopically visible area of discolouration. In addition, we investigated (ii) the location, structure and temporal appearance of spermogonia and aecia, and (iii) whether plant cellular damages and ultrastructural changes indicating an ongoing defence response can be detected following infection. The comparison of a resistant genotype with susceptible Norway spruce genotypes also allowed us to verify (iv) the presence of fungal hyphae in needles of the resistant tree and (v) anatomical–biochemical differences between the genotypes. Results can provide a better understanding of the biology of the fungus and its interaction with the plant, which is crucial to improve the selection of adapted Norway spruce seed accessions, varieties and genotypes, and their targeted propagation for afforestation projects.

## Materials and methods

### Sampling design

Needles were sampled from three susceptible and one highly resistant (PRA-R) adult Norway spruce tree growing side by side in a typical subalpine spruce forest in Praxmar, Tyrol, Austria (1614 m above sea level, N 47°09.495, E 11°08.201). The site is situated close to the natural tree line and the trees are highly affected by needle rust infection year after year due to high numbers of basidiospores from rhododendrons growing nearby. In the year of the experiment, an average of 49% of all current-year needles of the susceptible trees showed clear symptoms of rust infection, while the resistant PRA-R had only 4% symptomatic needles. PRA-R is the most rust resistant spruce genotype so far detected (Mayr et al. 2001, 2010, Ganthaler et al. 2017, Trujillo-Moya et al. 2022).

Sampling of the susceptible genotypes was started on 30 June, when the needles of the current year unfolded, started to elongate and showed the first yellow spots and continued several times (11 July, 28 July, 12 August 2022) until the trees started to shed the infected needles. Repeated sampling allowed all stages of infection and associated processes to be captured. Symptomatic needles from the resistant tree PRA-R and non-symptomatic needles from all trees were sampled at the last two time points for comparison. For each sampling, three current-year shoots were cut from different parts of the tree canopy, and from individual infected needles, a segment containing both a non-symptomatic (green) and a symptomatic (yellow) part was prepared (Figure 1E). These segments, ~5 mm long, were immediately stored and fixed in Eppendorf tubes filled with either 4% formaldehyde in phosphate buffer or modified Karnovsky solution (2.5% glutaraldehyde and 2% paraformaldehyde in cacodylate buffer).

### Histology

Before the paraffin embedding process, images were taken from fixed needles, both with and without infection symptoms (12 per time point, genotype and symptomatology). Affected needles were marked by a razor blade incision at the border between green (non-symptomatic) and yellow (symptomatic) areas. These marked needles were then paraffin embedded (Tissue-Tek VIP6, Sakura Fine Tek, Germany, Umkirch) RNA-Seq-night. Afterwards, 2.5 μm thick sections were cut longitudinally (over a yellow/green border) or transversely (in symptomatic and in non-symptomatic regions). Several serial sections were placed on a glass slide and heated before staining.

Haematoxylin and eosin staining (according to Mulisch and Welsch 2015) was used for a general evaluation of the morphology of symptomatic and non-symptomatic needles. Paraffin sections (2.5 μm) of selected needles from each tree were analysed for starch and neutral carbohydrates with periodic acid–Schiff staining (PAS, staining kit, Morphisto GmbH, Germany, Offenbach) according to the manufacturer’s protocol. The same set of needles was stained with Johansen’s quadruple stain (Johansen 1939) and toluidine blue. For the Johansen’s quadruple stain, sections were dewaxed with xylene and brought over a decreasing ethanol series to 70% ethanol. Afterwards, the sections were incubated for 25 h in a solution of 1 g safranin O with 50 mL ethylene glycol monomethyl ether, 25 mL 96% ethanol, 1 g natrium acetate and 4 mL 37% formaldehyde solution. The slides were then washed with tap water and incubated in 1% methyl violet solution. This quadruple staining enabled to distinguish between cell wall (blue), tannin (dark red), starch granules (red) and fungal hyphae (green). For the toluidine blue staining, the sections were incubated in 0.05% aqueous toluidine blue O (Merck, Darmstadt, Germany) solution for 20 min without prior dewaxing. Slides were then rinsed briefly with distilled water and 100% ethanol, dewaxed in xylene twice for 3 min and coverslipped with DPX medium (Merck). Toluidine blue stained the cell walls blue and tannin droplets yellow/green.

### Autofluorescence

Plants are known to contain a wide spectrum of autofluorescent molecules that can be useful for assessment of biochemical alterations (see review in Donaldson 2020). Paraffin sections were mounted on glass slides, dewaxed with xylene, rehydrated in a decreasing ethanol series and PBS, counterstained with DAPI (Merck) and coverslipped with Aqua-Polymount (Polysciences, Hirschberg, Germany). Autofluorescence was recorded for these sections in four channels (blue, green, orange, far red) with a fluorescence slide scanner (Evident Slideview VS200, Tokyo, Japan).

### P-phenylenediamine staining

Semi thin tissue sections (2 μm) of resin-embedded spruce needles were used for P-phenylenediamine (PPD) staining for lipid assessment. PPD 0.3 g (Merck) was dissolved in 30 mL ethanol absolute. Semi thin sections were stained for 5 min in the solution, afterwards washed by distilled water (three times 2 min each) and mounted with Aquatex (Merck).

### Transmission electron microscopy (TEM)

Immediately after collecting the needles, the basal part and the tip of the needle were removed for better infiltration of the fixative (2% paraformaldehyde/2.5% glutaraldehyde, Fluka Chemie GmbH, Buchs, Switzerland/Merck, Darmstadt, Germany) in cacodylate buffer (0.1 M, pH 7.4, Morphisto, Offenbach, Germany). In the histology laboratory, a segment that consisted of a non-symptomatic green part and a yellow symptomatic part was cut out of each needle. Before embedding, an incision exactly at the border between the green and yellow part was made with a razor blade in each symptomatic needle. One segment per non-symptomatic needle (green) was taken.

After washing three times in cacodylate buffer, samples were postfixed for 2 days in a 1% solution of osmium tetroxide (Electron Microscopy Sciences, Hatfield, USA) diluted in cacodylate buffer at room temperature and after that, washed in cacodylate buffer again. Dehydration was performed in a series of graded ethanol solutions (70%, 80%, 96% and 100%). Subsequently, samples were infiltrated with propylene oxide (Merck), followed by increasing ratios of epoxy resin–propylene oxide and finally pure resin (Serva, Mannheim, Germany). After an additional change, the resin was polymerized at 60 °C for 24 h. Semi thin sections were cut at 0.8 μm and stained with toluidine blue for general assessment and orientation. Ultra-thin sections were cut at 70 nm, mounted on copper grids (Science Services, Munich, Germany) and stained with uranyl acetate (Fluka Chemie GmbH, Buchs, Switzerland) and lead citrate (Merck). Transmission electron images were made with an EM900 (Zeiss, Oberkochen, Germany).

Specific parts of paraffin embedded symptomatic needles of resistant trees were punched out of the block and re-embedded in Epon resin (Serva, Mannheim, Germany) for TEM analysis. In brief, paraffin was removed by xylene and samples were rehydrated in a series of graded ethanol solutions (100%, 96%, 80%, 70%) and washed in cacodylate buffer. Samples were then postfixed with 2% paraformaldehyde/2.5% glutaraldehyde, osmicated for 24 h in 1% osmium and afterwards embedded in Epon.

## Results

### Fungal structures and host–pathogen interaction in the needle tissues

**Figure 2:**
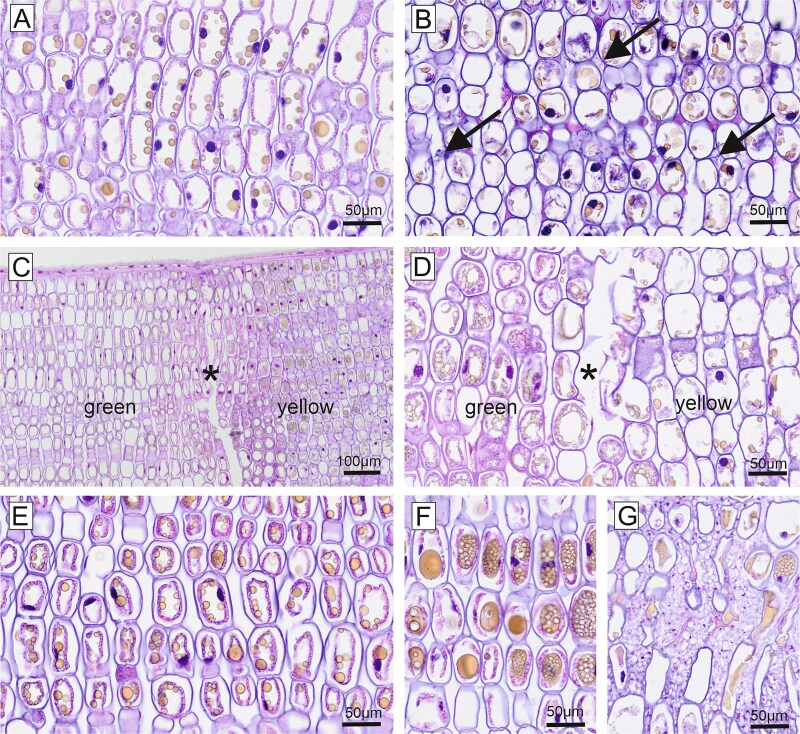
Longitudinal paraffin sections of Norway spruce needles from a susceptible (A–D) and the resistant tree (E–G), HE staining. (A) Non-symptomatic spruce needle of susceptible tree. Ring-shaped pink cytoplasm with chloroplasts, blue cell wall, dark blue nuclei and yellowish tannin droplets; intercellular space was clear. (B) Symptomatic needle of susceptible tree. Cytoplasm degraded, nuclei and tannin droplets disorganized; intercellular space occupied by fungal hyphae (arrows). (C) Border between green and yellow part of a symptomatic needle was marked by scratch before embedding (asterisk). A clear difference between the healthy green part and the damaged yellow part was observed. (D) Larger magnification showed degenerated cytoplasm in the infected part compared with the healthy part. (E) Non-symptomatic needle of resistant tree. Cytoplasm, cell wall, nuclei and tannin droplets were perfectly organized. Large chloroplasts and tannin droplets. (F) Symptomatic needle of resistant tree showed partly extremely enlarged tannin droplets and granular yellowish central vacuoles. In rare cases also fungal hyphae were present between cells, intermingled with intercellular material (G).

**Figure 3:**
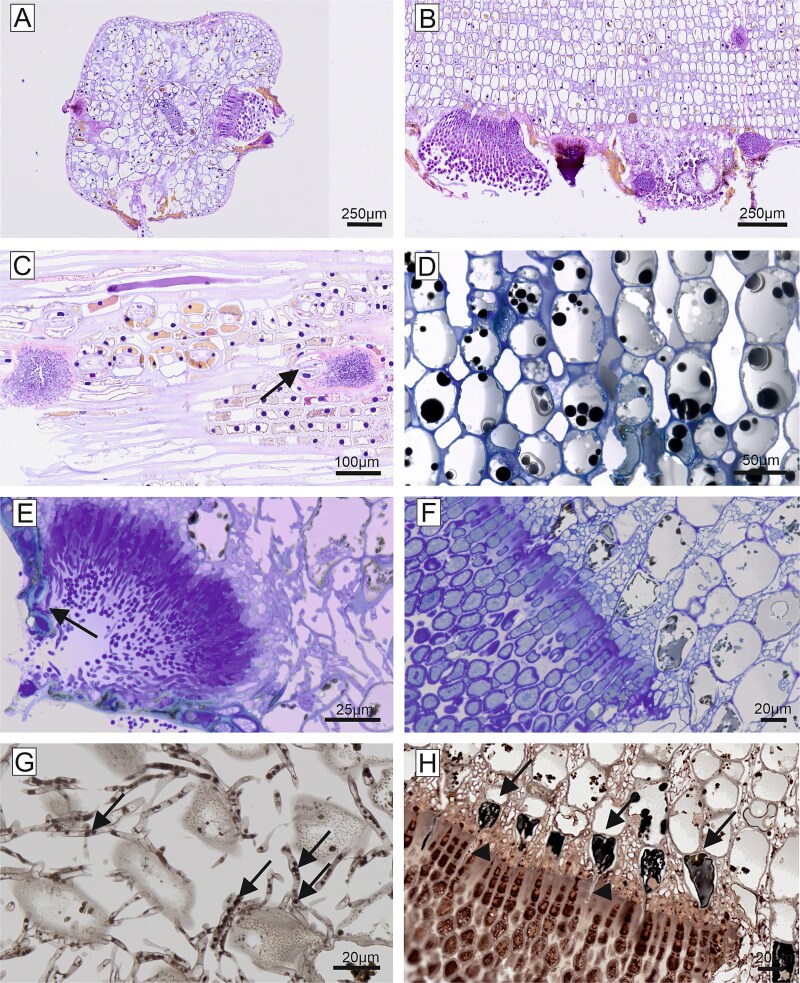
Paraffin sections, HE staining (A–C). Semithin resin sections, toluidine blue staining (D–F). Semithin resin sections, PPD staining (G, H). (A) Symptomatic needle of susceptible tree with two fully developed aecia (A) and one spermogonium (S). The epidermis was stained in orange and had broken open in the area of the aecia. (B) Longitudinal section of larger aecia and smaller spermogonia. Note that spermogonia were partly located very close to aecia. (C) Longitudinal HE-stained section with stomata and spermogonia. Spermogonia were arranged within the stomata rows and opened beside a stoma (arrow). (D) Semithin section of symptomatic needle from resistant tree showing intercellular material (arrow) and large tannin granules (black). (E) Resin section of spermogonium in a symptomatic needle of a susceptible tree with underlying network of fungal hyphae. Opening and release of spores beside stoma (arrow). (F) Aecia in a symptomatic needle of a susceptible tree with underlying large plant cells with grey/black tannin content. Aeciospores separated from chains of elongated cells. Network of fungal hyphae spreads below aecia. (G) PPD staining showed a high amount of lipid droplets (arrows) within fungal hyphae (dark brown). (H) Aecia with dark brown spores indicating a high lipid content in symptomatic needle of a susceptible tree. Note the tannin filled, black, large plant cells below the aecia base (arrows). These large cells had protrusions (arrowheads) in the direction of the spore layer.

**Figure 4:**
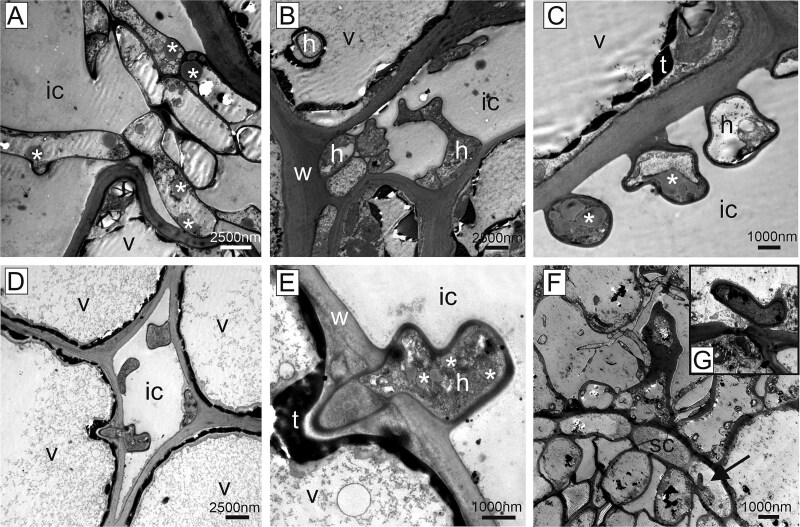
Ultrathin sections, transmission electron microscopy of fungal structures. Symptomatic needles of susceptible trees were characterized by fungal hyphae invading the intercellular space. Fungal hyphae were often provided with lipid droplets (asterisks) and adhered to the plant cell wall (A–D). (D, E) Occasionally, the fungus was observed to penetrate the cell wall by forming an arrow-shaped penetration peg. (F) In rare cases, also the endodermis cells of the vascular bundle were invaded by fungal hyphae (arrow—for higher magnification see inset g). Cell wall (w), intercellular space (ic), fungal hypha (h), endodermis (sc), tannin (t), vacuole (v).

Infection with *C. rhododendri* in symptomatic needles of susceptible spruce trees manifested as externally visible spermogonia, aecia and tissue discoloration, and internally growing mycelium and related plant cellular changes. While in the intercellular space of symptomatic needles fungal mycelium was frequently observed, non-symptomatic needles were completely fungus free (Figure 2A and B). Longitudinal histological sections of symptomatic needles, spanning over the externally visible border between symptomatic (yellow) and non-symptomatic (green) needle parts (scratch marked) demonstrated that the green/yellow colour change exactly reflected the fungal mycelial invasion front and associated cellular alterations (Figure 2C and D). Spermogonia (pycnia) were subepidermal and characterized by a triangular shape with the hymenial layer originating from the base (Figure 3A and B). On the upper side, towards the narrow opening, round to oval spermatia were seen. Spermogonia were regularly located along the stomata rows of the spruce needle, and the openings were always located next to a stoma (Figure 3C and E). Aecia were also subepidermal in origin and were covered by a peridium; they led to a bursting of the needle epidermis. Aecia were usually preceded or accompanied by spermogonia and both were regularly observed very close together and even intermingled (Figure 3A and B). Fungal hyphae were rarely seen intracellularly in symptomatic needles but spread mainly between adjacent mesophyll cells and connected the spermogonia and the aecia (Figure 3E and F). Hyphae as well as both types of fungal spores (spermatia and aeciospores) were characterized by numerous lipid droplets, visible at the light microscopic level by PPD staining of Semi thin resin sections (Figure 3G and H). Directly below the aecia, plant mesophyll cells with a high tannin content, stained black in resin sections by osmium postfixation, were abundant. These cells had long protrusions towards the aecium. Plant cell wall material was abundant between the adjacent aeciospores (Figure 3H). On the ultrastructural level, it was observed that membranes of fungal hyphae were tightly attached to the outer host cell wall by some kind of connective/adhesive material (Figure 4A–E). Fungal hyphae were observed to penetrate the cell wall of host cells by forming a penetration peg at the tip of the hyphae (Figure 4D and E). Consequently, fungal hyphae were observed also intracellularly within mesophyll cells, covered by cytoplasm and surrounded by electron dense (*tannin*) material (Figure 4B–D). The fungal invasion also reached the endodermis surrounding the central vascular cylinder (Figure 4F).

**Figure 5:**
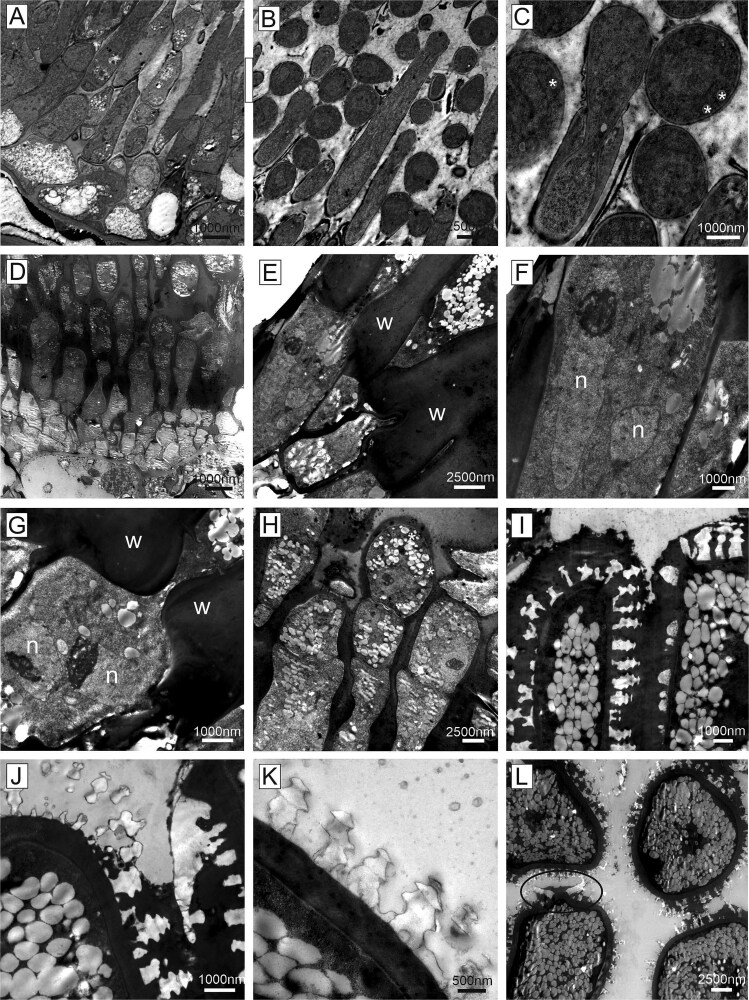
Ultrathin sections, transmission electron microscopy. Spore development in spermogonia (A–C) and aecia (D–L) in symptomatic needles of susceptible trees. Spermogonia consisted of elongated hyphae with spores released from the tips (B and C). Spores contained lipid droplets (asterisks). (D, G, H) Aecia were built of chains of binucleate cells with spores separating at their distal end. (E–G) Thick plant cell wall remnants were present between spores. (H) Lipid droplets were present in proximal cells, but their amount increased significantly in more developed spores. (I, J) Spores were covered by white warts and a layer of electron dense material that embedded the warts. (K) Fully developed spores carried free warts and were filled with masses of lipid droplets. (L) A cap with two larger, antenna-like warts was frequently observed at both ends of aeciospores (encircled). Cell wall (w), nucleus (n).

Spermogonia and aecia differed greatly at the electron microscopic level. Spermatia from spermogonia were round to oval shaped, small (average size 3 μm) and developed from chains of elongated cells (Figure 5A–C). Spermatia had a cell wall, a homogenous internal appearance and contained a limited amount of lipid droplets (Figure 5C). Aecia were characterized by larger complexes and aeciospores were also much larger (average size 14 μm) than spermatia, arranged in chains, binucleate and contained increasing masses of lipid droplets as development progressed (Figure 5D–L). No intercalated cells were observed. The aeciospores underwent a progressive morphological transition (representing the maturation) during their formation (Figure 5I–L). The spores at the base of the aecia were covered with a layer of cell wall material in which a high number of white, angular processes (warts) were embedded. In more mature spores, this layer gradually disappeared, and the angular white warts now covered the spore surface. In addition, the aeciospores had caps at their ends with pairs of longer warts (Figure 5L).

In the tree with enhanced resistance, fungal hyphae were also observed in the intercellular area of symptomatic needles (Figure 2G), but in much lower numbers than in susceptible trees. Needle cells of the resistant tree were normally organized, but characterized by large tannin droplets (Figure 2E and F) and the intercellular space was frequently occupied by a grey material even if no fungal hyphae were present (Figure 3D).

### Infection-caused changes in the structure of host cells

**Figure 6:**
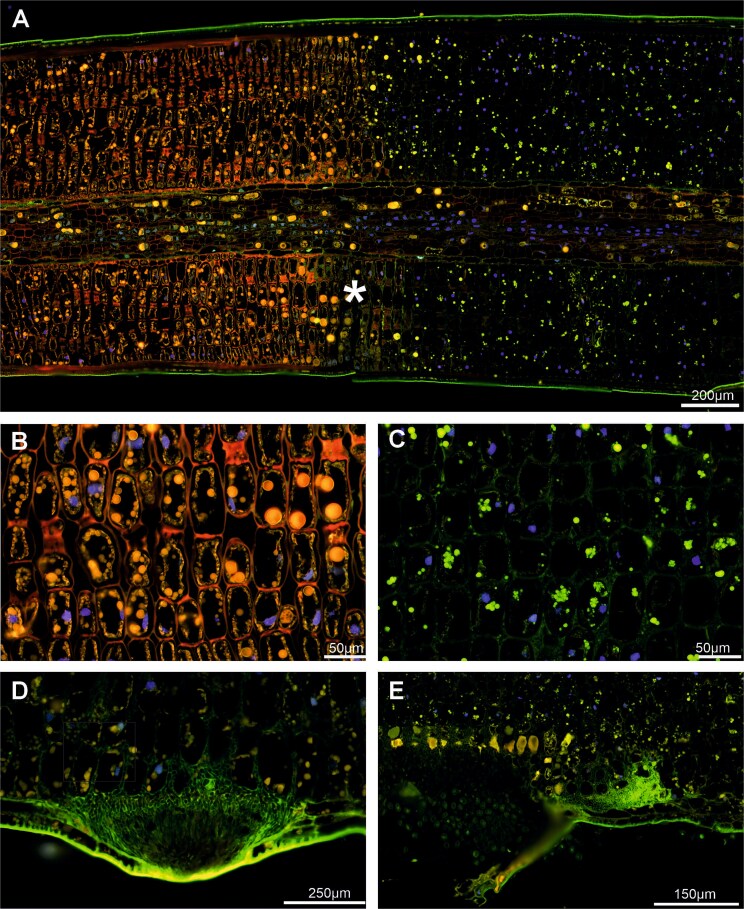
Longitudinal paraffin sections of symptomatic needle of susceptible tree, autofluorescence. (A) Scratch-marked border (asterisk) separated the non-symptomatic needle part with red/orange fluorescence (representing chlorophyll, tannin and cell wall components) from the symptomatic needle part which completely lost this emission range. (B, C) Larger magnification showing the morphological difference between non-symptomatic and symptomatic part. (D) Spermogonia and fungal hyphae had a green autofluorescence. (E) Aeciospores only partly emitted green light. Large plant mesophyll cells, frequently present below aecia, emitted orange light (tannin).

Autofluorescence analyses with scratch marked needles (border between yellow and green needle part) showed a clear demarcation of different light emission spectra in non-symptomatic and symptomatic needle parts (Figure 6A–C). Whereas in the non-symptomatic, green parts of the needle, the cell walls of the mesophyll cells emitted red, the part colonized by the fungus completely lost the emission of this wavelength and the cell walls appeared in green (weak signal). The orange fluorescence of the plastids and tannin droplets was completely lost in the symptomatic part, only some green fluorescing droplet-like particles were present (Figure 6A–C). Yellow/orange signals were mostly seen in enlarged mesophyll cells below the aecia (Figure 6E). The spermogonia, the fungal mycelium and spores presented in green (Figure 6D and E).

HE-stained paraffin sections of symptomatic and non-symptomatic Norway spruce needles of susceptible trees also revealed clear morphological differences (Figure 2). In longitudinal sections of non-symptomatic needles, palisade parenchyma cells (mesophyll) with a regular, ring-shaped cytoplasm with chloroplasts around a central vacuole were present. Nuclei were integrated into the ring-shaped cytoplasm, as were yellowish round tannin droplets of various sizes. The intercellular space was completely clear (Figure 2A). In symptomatic needles, the cell walls were intact, but the intracellular morphology was significantly altered. The cytoplasm was disintegrated, the central vacuole was not clearly demarcated and contained yellowish, clumped, irregularly shaped tannin material of various sizes (Figure 2B). In the resistant tree, mesophyll cells of non-symptomatic needles showed a clear cytoplasmic ring with chloroplasts and a high number of yellowish round tannin droplets of various sizes (Figure 2E). In symptomatic needles, the tannin droplets were enlarged and predominantly aggregated in the cell centre, partly with a foamy/granular appearance (Figure 2F).

**Figure 7:**
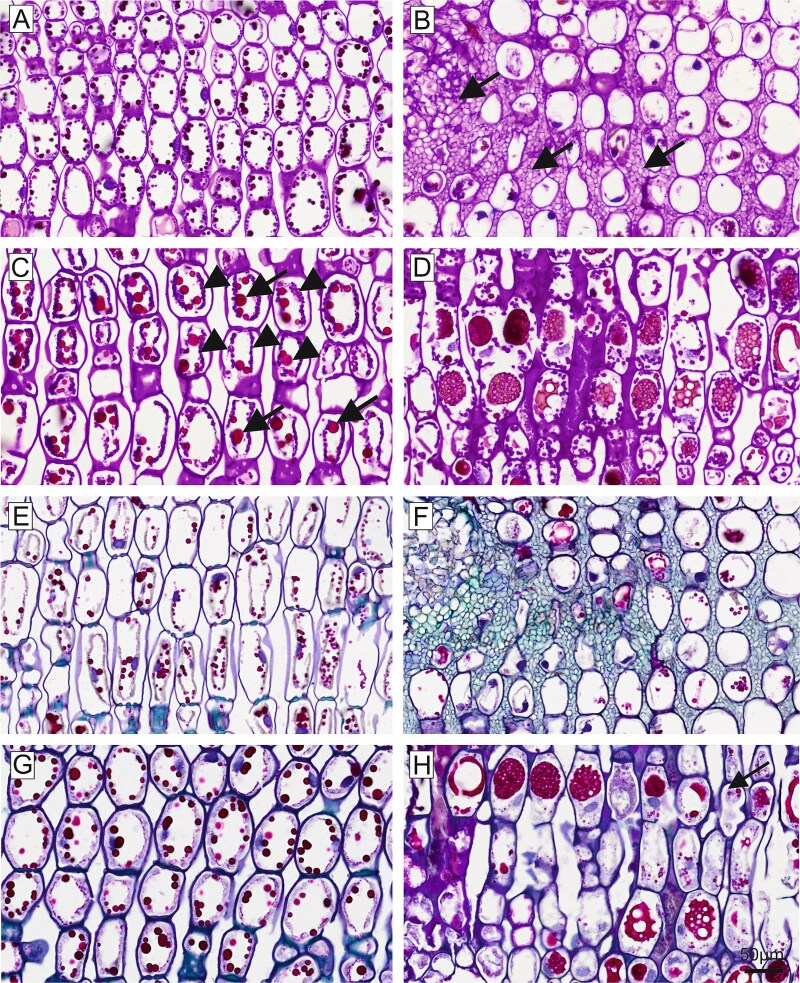
Longitudinal paraffin sections, PAS staining (A–D); Johansen quadruple staining (E–H). (A) Non-symptomatic needle of susceptible tree with PAS-positive stained cell walls, chloroplasts and tannin droplets. (B) Symptomatic needle of susceptible tree with PAS-positive cell wall and fungal hyphae in the intercellular space. (C) Non-symptomatic needle of resistant tree showed PAS-positive cell walls, small starch granules in the cytoplasm (indicated by arrow heads), chloroplasts and tannin as large, bright red granules (indicated by arrows). (D) Symptomatic needle of resistant tree. Beside cell walls, the inner vacuole contained PAS-positive material. In addition, the intercellular space was filled with PAS-positive material. (E) Non-symptomatic needle of susceptible tree with blue stained cells walls, nuclei and red-stained tannin droplets. (F) Symptomatic needle of susceptible tree with blue stained cell walls, some blue stained nuclei and red stained remnants of cytoplasm. Fungal hyphae in the intercellular space were stained in green. (G) Non-symptomatic needle of resistant tree. Cell walls stained in blue, large tannin droplets in red and small starch granules in the cytoplasm in pink. (H) Symptomatic needle of resistant tree. Cell walls were stained in blue; remnants of red granules were present in the cytoplasm. Note that the central vacuoles and the intercellular space were partly filled with red foamy/granular material.

Differences in morphology between symptomatic and non-symptomatic needles of susceptible trees were observed also by PAS staining of histological sections (Figure 7). Mesophyll cell walls and cytoplasmic starch granules in non-symptomatic needles appeared magenta after PAS staining, indicating a high content of neutral carbohydrates. In addition, dark red stained droplets were located along the cytoplasmic ring, most likely representing tannin granules (Figure 7A). In symptomatic needles, the cell walls were still PAS-positive, but only remnants of PAS-stained structures were left in the degraded cytoplasm. Intercellular hyphae were strongly PAS-positive (Figure 7B). Non-symptomatic needles of the resistant tree were characterized by significantly larger magenta-coloured granules in the cytoplasm and larger, bright-red tannin droplets (Figure 7C). In symptomatic needles of the resistant tree, the cytoplasm was degraded, but starch granules and some tannin droplets were still present. In addition, the central vacuole contained a foamy/granular, red-stained material and a strongly pink-stained amorphous material was locally present between the cells (Figure 7D).

The quadruple staining after Johansen resulted in non-symptomatic needles of susceptible trees in red staining of tannin droplets and starch granules in the cytoplasm, blue staining of cell nuclei, violet staining of mesophyll cell walls and cyan staining of the middle lamellae (Figure 7E). In symptomatic needles, only remnants of red stained granules and blue cell nuclei were seen. Fungal mycelium was stained green and occupied the intercellular space (Figure 7F). Again, non-symptomatic needles of the resistant tree had larger red tannin droplets than non-symptomatic needles of susceptible trees (Figure 7G). These granules almost disappeared in symptomatic needles of the resistant tree; however, a red-stained, foamy/granular material in the central vacuole was present in many cells. The intercellular material presented in blue and red (Figure 7H).

**Figure 8:**
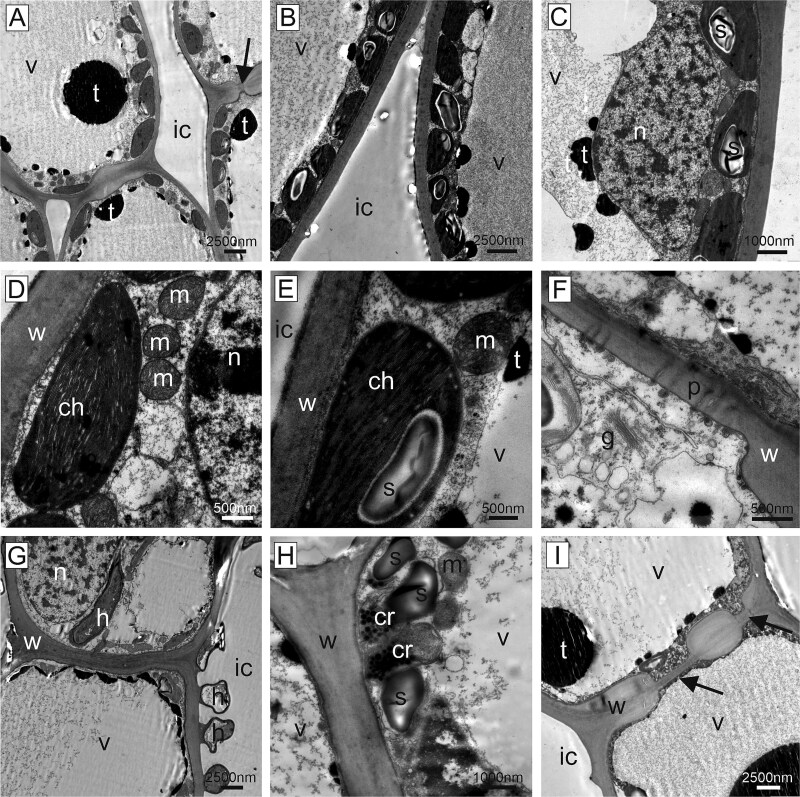
Ultrathin sections, transmission electron microscopy. Non-symptomatic needles of susceptible trees (A–F) were characterized by homogenous cell walls, plasmodesmata and clear intercellular spaces. The cytoplasm was arranged along the cell wall containing chloroplasts with starch granules (s) and all cell organelles. The central vacuole appeared clear or filled with fine granulated material. Tannin was present in the form of black round droplets and fine granules. Symptomatic needles of susceptible trees (G–I) showed a degenerated cytoplasm with some remnants of chloroplasts and starch granules. Plasmodesmata (arrows) were still present, tannin was present as large and small droplets or as a layer along the central vacuole. Cell wall (w), intercellular space (ic), chloroplasts (ch), chloroplast remnants (cr), fungal hypha (h), Golgi apparatus (g), nucleus (n), starch granule (s), mitochondrion (m), plasmodesmata (p), tannin (t), vacuole (v).

**Figure 9:**
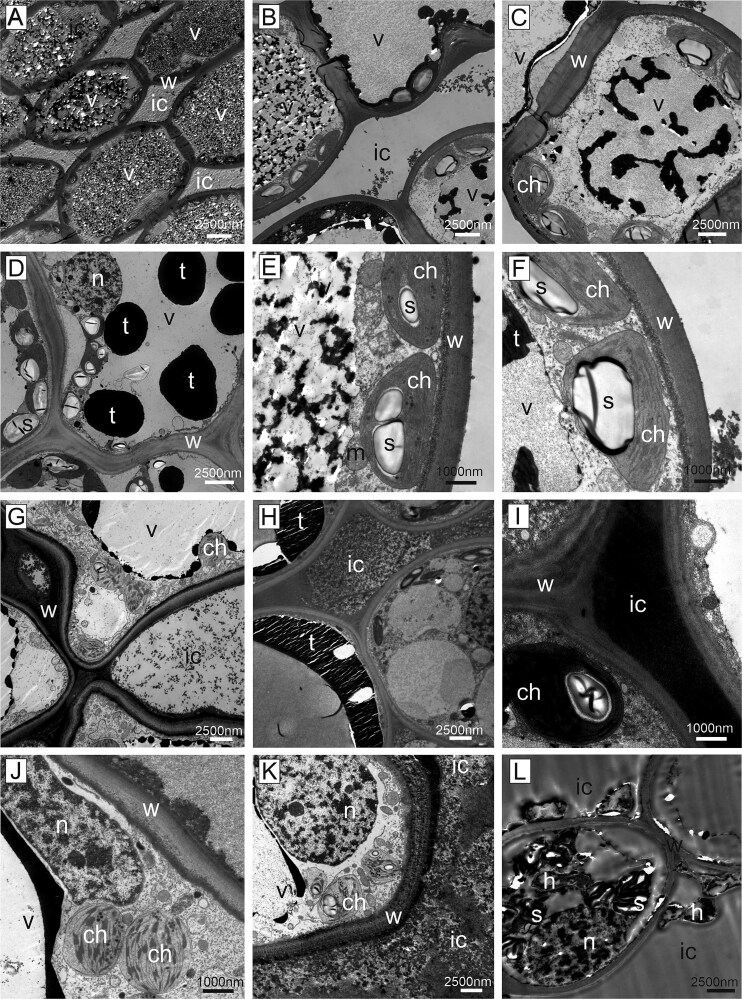
Ultrathin sections, transmission electron microscopy. Non-symptomatic needles (A–F) and symptomatic needles of resistant tree (G–L). (A) Longitudinal sections of non-symptomatic needles showed characteristic filled central vacuoles. (B–F) The vacuoles contained electron dense material (tannin) either in large droplets, or granular and clumped structure. The cytoplasm was comparable to non-symptomatic needles of susceptible trees, with normal cytoplasm, demarcated vacuole and chloroplasts. Starch granules tended to be larger. (G–L) Symptomatic needles contained various materials in the intercellular spaces, from fine granular (g) to fully electron dense material (i). While the cytoplasm with the organelles was perfectly intact, the tannin distribution changed from droplet form to a layer of electron dense material between the central vacuole and the cytoplasm. The plant cell wall changed its structure from homogenous to layered and granular. Fungal hyphae were present in the intercellular space and occasionally intracellularly (l). Cell wall (w), intercellular space (ic), chloroplast (ch), fungal hypha (h), mitochondrion (m), nucleus (n), starch granule (s), tannin (t), vacuole (v).

Epoxy-resin sections of symptomatic and non-symptomatic needles of susceptible trees observed by transmission electron microscopy revealed clear ultrastructural differences (Figure 8). The mesophyll cells of non-symptomatic needles were characterized by a regular cytoplasm located along the homogenous cell wall and contained chloroplasts with starch granules and other cell organelles such as mitochondria, Golgi apparatus and cell nuclei (Figure 8A–F). Adjacent cells were connected by plasmodesmata, and the intercellular space was clear. Occasionally, round electron dense droplets (tannin) were present in the central vacuole, and smaller droplets were arranged along the cytoplasm (Figure 8A). In symptomatic needles of susceptible trees, the cytoplasm of host cells was largely degraded. Some remnants of degenerated organelles such as chloroplasts, some with starch granules, were occasionally still visible. The demarcation between the central vacuole and the cytoplasm was faint, but the cytoplasm was covered in this area with a layer of electron dense material (Figure 8G). In the resistant tree, the central vacuole of non-symptomatic needles was occupied by electron dense granular material (tannin). This material appeared quite variable, either as large droplets, or granular and clumped within the central vacuole (Figure 9A–F). The cytoplasm was arranged in a ring-like pattern, as in susceptible trees, but starch granules tended to be larger (Figure 9E and F). In the mesophyll cells of the rare symptomatic needles of the resistant tree, cellular organelles were not degraded, and all components were present as in non-symptomatic needles (Figure 9G–L). However, the tannin content was rearranged from a droplet shape to a layer of electron dense material, covering the entire cytoplasm and forming a kind of border between the central vacuole and the organelles (Figure 9G–K). The intercellular space in symptomatic needles of the resistant tree was occupied by electron dense material (Figure 9G–I) and the cell wall had an altered appearance compared with non-symptomatic needles of this tree (Figure 9K). Fungal hyphae were observed in the mesophyll inter- and intracellular space rather rarely (Figure 9L). Under the epidermis, fungal outwards protrusions in the host cell wall were regularly found in the symptomatic area.

## Discussion

Due to their obligate biotrophy and complex life cycle with host shift and multiple spore types, rusts are difficult organisms to study (Staples 2000, Lorrain et al. 2019). This is particularly true for species from high-mountain environments that attack long-living woody species, such as *C. rhododendri*, where both field studies and controlled experiments are challenging. Given that the first part of the life cycle in the telial host, rhododendrons, has been reported previously (Berndt 1999), and ecological and economic damages mainly result from damage to the aecial host Norway spruce (Ganthaler et al. 2014), we focused here on the fungal development in spruce needles and ultrastructural differences between susceptible and resistant trees.

### Fungal development in the needle

*Chrysomyxa rhododendri* spread, after the entering of the spruce needle, primarily intercellularly in the host tissue (Figures 4A–C and 7B and F). The infection hyphae grew from the substomatal intercellular space towards the mesophyll cells, making it likely that the rust entered the needles via or next to the stomata (cf. Harder 1984, Jurgens et al. 2003, Kolmer et al. 2009). Previous analyses suggested that stomata closure, which could prevent the pathogen entry, may be hindered by low abscisic acid levels and blockage of the Ca^2+^ signalling pathway in susceptible spruce genotypes (Trujillo-Moya et al. 2020, 2022). The hyphae were partly attached to the host cell walls, and, in later stages, began to invade the living mesophyll cells with a penetration peg (Figure 4D–F). The tip of the peg was delimited by a septum and is thought to form the haustoria within the host cell, a specialized structure of rusts to take up nutrients from the host cell without destroying it (Harder 1984, Staples 2000). The haustoria are required as obligate parasites depend on nutrients from living cells (Kolmer et al. 2009, Lorrain et al. 2019) and have been previously described for several *Chrysomyxa* species (Berndt 1999). Fungal hyphae were also observed in the needle endodermis (Figure 4F) but never in the vascular system like reported, for example, for white pine blister rust (Jacobs et al. 2009), which is consistent with the observation that *C. rhododendri* does not spread to the branches or between needles (Ganthaler et al. 2017).

After developing a dense mycelium in the intercellular spaces and getting access to the host nutrients (Figures 2B and 4), *C. rhododendri* started to form spermogonia and then aecia in the spruce needle (Figure 1C). Spermogonia (pycnia) of this needle rust can be assigned, based on the histological images, to the morphological type Group I, Type II according to Cummins and Hiratsuka (1983), which is flat and located subepidermal (Figure 3E and F). Macroscopically they appear as small, exalted, brown dots along the stomata rows (Figures 1 and 3C and E). In the spermogonia, the plasmogamy occurs, i.e., haploid spermatia fuse with corresponding conception hairs to form a dikaryotic cell which is the basis for the aecia (Bennell 1985, Duplessis et al. 2021, McTaggart et al. 2022). The round to oval shaped spermatia of *C. rhododendri* were released in an exudate in large numbers from spermogons in the spermogonia (Figures 3E and 5A–C). Subsequently aecia were formed, distinctly larger than the spermogonia, subepidermal in origin, erumpent and with peridium (Figure 3B and F). Within the aecia the different developmental stages of spore formation were well visible (Figure 5D–L), leading finally to the verrucose aeciospores. Aeciospores were morphologically very similar to the basidiospores and urediniospores of the species (Berndt 1999, Ganthaler and Mayr 2015), with characteristic warts on the surface and small caps at their ends (compare also Crane 2001). Spores were also filled with large amounts of yellow lipid droplets, probably containing β- and γ-carotene (Pfeifhofer 1989). The spores were released by disruption of the aecial peridium and the needle cuticula and complete the life cycle of the rust by infecting again the telial host rhododendrons (Bennell 1985, Duplessis et al. 2021).

No fungal cells were detected in non-symptomatic needles (Figures 2A, 7A and E, and 8A), demonstrating that healthy-appearing needles of infected trees are *C. rhododendri*-free and can thus serve as controls in physiological and molecular studies (e.g., Ganthaler et al. 2017, Trujillo-Moya et al. 2022). In addition, the growth of the fungal mycelium was restricted to those parts of the needle that appeared macroscopically as yellow stripes (Figures 2 and 6) and led to associated, also locally restricted, cell damage and defence responses. Exact correlation of symptom development with mycelial growth and distribution has also been shown for other rusts, such as *Puccinia hordei* on barley (Scholes and Farrar 1987).

### Infection-induced host cellular changes

The way in which the fungus enters and colonizes the host tissue is highly relevant for the type and extent of damage caused (Doehlemann et al. 2017). Intracellular biotrophic pathogens, such as *C. rhododendri*, generally adversely affect the plant to a higher extent than fungi that do not form close contact with the host cell, but at the same time try to keep the cells alive.

Host cells in the symptomatic needle area showed extensive degradation, including degeneration of the cytoplasm and the chloroplasts (Figures 2B and 8G–I), in contrast to non-symptomatic needles and non-symptomatic needle parts, which showed a ring-shaped cytoplasm along the cell wall with evenly arranged chloroplasts and a completely clear intercellular space (Figures 2A and 8A–F). Previous physiological studies had already shown a chlorophyll breakdown and impaired chloroplast function in needle rust infected trees, as well as an under-expression of many genes involved in photosynthesis, suggesting a degradation of the chloroplast and disturbances in the functioning of several cell processes and metabolic pathways (Bauer et al. 2000, Mayr et al. 2001, Trujillo-Moya et al. 2020). The typical yellowing of attacked needle parts, which is clearly visible in midsummer in affected forest areas (Figure 1A and B), is also based on the reduced plastid pigment content as well as on the production of carotene by the fungus (Pfeifhofer 1989), which accumulates in large lipid droplets in both hyphae and spores (Figures 3G and H, 4 and 5K). The loss of red/orange autofluorescence in symptomatic needle parts (Figure 6) also points to the chlorophyll degradation, and in addition to modifications in the cell wall (Donaldson 2020). However, cells were not collapsing and plasmodesmata and tannin droplets were still present, the latter partly disorganized or arranged in a layer between the vacuole and the plasma membrane, possibly representing an attempt by the plant to inhibit the first step of the interaction between the fungus and the plant protoplast (Novacky 1991). The large plant cells with protrusions observed below the aecia (Figures 3H and 6E), which likely contain large amounts of polyphenolic compounds, could serve the same purpose and are consistent with reported changes in the phenolic profile following infection (Ganthaler et al. 2017, 2023). Interestingly, also cells at the border between chlorotic and healthy needle parts contained higher amounts of tannin droplets and a highly fluorescent cell wall (Figure 4), indicating an induced defence response of the plant.

Grill et al. (1978) reported for *C. abietis* a close contact of the hyphae with the nucleus, which we could not directly observe here, but a disorganization of the nuclei (Figures 2B and 8G–I). The few remnants of starch granules still visible (Figure 8H) explain the lower concentrations of non-structural carbohydrates found in symptomatic needles (only 60% of control healthy trees; Bauer et al. 2000). Starch reduction is mainly due to the reduced photosynthetic activity, but probably also to increased respiration and starch degradation (genes of key enzymes were reported to be overexpressed), and the uptake by the fungus (Plattner et al. 1999, Trujillo-Moya et al. 2020). In summary, in needles infected with *C. rhododendri,* we observed (i) direct damage caused by the fungus, such as the cell degeneration, (ii) indirect consequences due to reduced nutrients, such as smaller starch grains and (iii) induced defence responses by the plant, such as the accumulation of specific compounds in cells close to fungal hyphae as well as spermogonia and aecia. The resulting anatomical and physiological changes in the host tissue are probably the reason why spruce needles with extensive symptoms are shed by the tree in the same summer, as well as the tree’s attempt to get rid of the fungus (Bennell 1985). Needle shedding is a significant loss for the tree because *C. rhododendri,* like all rusts, parasitizes fresh tissue from vigorously growing plants, unlike several other plant pathogens which tend to attack weakened or poorly growing plants (Cummins and Hiratsuka 1983, Bostock et al. 2014).

### Characteristics of needles with enhanced resistance

Initial attempts to understand the underlying mechanisms of the extremely rare *C. rhododendri* resistant trees focussed on phenological or morphological needle differences, such as a thicker or more rapidly developing cuticula and epidermis, or variations in the stomatal anatomy that prevent germ tube entry (Bauer and Schwaninger 2007, Mayr et al. 2010). As no evidence has been found for this, recent studies have investigated the spruce’s molecular defence, suggesting a strong HR in resistant trees, associated with the accumulation of secondary compounds (Trujillo-Moya et al. 2020, 2022). Based on the histological and TEM images presented here, we can confirm that *C. rhododendri* has been able to invade the needles of the resistant tree PRA-R, but the growth and spread within the needles appeared to be strongly restricted (Figure 2G). Spermogonia and aecia were also very rare, probably because the fungus had limited access to the required plant’s nutrient resources (Voegele and Mendgen 2003). Accordingly, infection-induced damage in the resistant tree was low, and in most mesophyll cells, the organelles were normally arranged and contained large chloroplasts and starch grains as in healthy needles (Figures 2E and 9F).

However, specific differences in needle cell characteristics in resistant compared with susceptible trees appeared, both in non-symptomatic needles (representing a constitutive defence) and in symptomatic needles (induced defence), which provide important indications on the underlying resistance mechanisms. Firstly, in resistant needles, the accumulation of intercellular material was observed (Figures 3D and 9B). This phenomenon was even stronger when needles were symptomatic, as then various materials, from fine granular to fully electron dense, filled the intercellular spaces (Figure 9G–I). As images also indicated granular material in the vacuoles, a modification of the cell wall (from homogenous to granular and with distinct layers) and the secretion of material on their surfaces (Figure 9I–K), results may indicate an infection-induced enhanced production and incorporation of secondary compounds, particularly polyphenols, in the cell walls and their deposition in the intercellular spaces. It was only recently shown for Norway spruce that extracellular vesicles can carry lignin precursors to the apoplast (Kankaanpää et al. 2024) and lignification is an important factor in the HR and resistance to rust (Beardmore et al. 1983, Bonello and Blodgett 2003). This may not only create chemical barriers and inhibit the spread of the pathogen (Matern and Kneusel 1988, Jurgens et al. 2003, Ullah et al. 2017) but also hinder penetration of plant cell walls via the inactivation of fungal pectic enzymes by the polyphenols (Lattanzio et al. 2006). Secondly, needle mesophyll cells of the resistant tree contained larger tannin droplets and granules in the vacuole than needles of the susceptible trees (Figures 2D, 3D, 7C and G, 9C and D) and, following infection, they were partly extremely enlarged and formed a broad layer between central vacuole and plasmalemma (Figures 2F and 9). Higher production of tannin, a group of flavonoids, was mainly associated with herbivory but also with fungal resistance (Brignolas et al. 1995, Treutter 2006, Zhu et al. 2019), can be induced by defence-related phytohormones (Iqbal and Poór 2025) and was similarly reported also for white pine infected by the rust *Cronartium ribicola* (Jacobs et al. 2009).

Both observations underline that the resistance is based on a combination of constitutive higher defences (i.e., accumulation of high defence compound levels in healthy needles, e.g., in the vacuole) with a fast induced response (i.e., modification and translocation of compounds to the cell walls and intercellular spaces in case of infection) (Cvikrova et al. 2006, Sampedro 2014). Observed higher tannin content and modification of the cell wall are also in agreement with previous results gained by molecular and biochemical analyses that indicated changes in the phenolic profile (Ganthaler et al. 2017), and an effective HR in the resistant tree (Trujillo-Moya et al. 2022).

## Conclusion

This study significantly expands our knowledge of the biology of the rust species *C. rhododendri*, which like several other rusts that attack forest tree species (Hamelin 2022), is still poorly understood, and of the associated plant defence responses. Both aspects are essential to better understand the physiological limitations of symptomatic trees and have important implications for the search for pathogen control strategies (Prell and Day 2001). Naturally occurring trees with enhanced resistance represent a promising approach (Naidoo et al. 2019), and the results of this study underline that these specimens cannot prevent the entry of the fungus into the needle, but are able to effectively limit mycelial growth and the formation of spermogonia and aecia. A key mechanism appears to be the reinforcement of plant cell walls and the accumulation and reorganization of secondary compounds such as tannins.

## Data Availability

All main results are presented within the manuscript and the Supplementary data. The raw data that support the findings of this study are available from the corresponding author upon reasonable request.
